# Dietary patterns are associated with cognitive function in the REasons for Geographic And Racial Differences in Stroke (REGARDS) cohort

**DOI:** 10.1017/jns.2016.27

**Published:** 2016-09-28

**Authors:** Keith E. Pearson, Virginia G. Wadley, Leslie A. McClure, James M. Shikany, Fred W. Unverzagt, Suzanne E. Judd

**Affiliations:** 1 Department of Nutrition Sciences, University of Alabama at Birmingham, Birmingham, AL, USA; 2 Department of Medicine, University of Alabama at Birmingham, Birmingham, AL, USA; 3 Department of Epidemiology and Biostatistics, Drexel University, Philadelphia, PA, USA; 4 Division of Preventive Medicine, University of Alabama at Birmingham School of Medicine, Birmingham, AL, USA; 5 Department of Psychiatry, Indiana University School of Medicine, Indianapolis, IN, USA; 6 Department of Biostatistics, University of Alabama at Birmingham, Birmingham, AL, USA

**Keywords:** Dietary patterns, Cognition, Nutrition, Cognitive function, AFT, Animal Fluency Test, Block98 FFQ, Block98 food frequency questionnaire, Q1, lowest quintile, Q5, highest quintile, PCA, principal component analysis, REGARDS, REasons for Geographic And Racial Differences in Stroke, SIS, Six-Item Screener, WLDR, Word List Delayed Recall, WLL, Word List Learning

## Abstract

Identifying factors that contribute to the preservation of cognitive function is imperative to maintaining quality of life in advanced years. Of modifiable risk factors, diet quality has emerged as a promising candidate to make an impact on cognition. The objective of this study was to evaluate associations between empirically derived dietary patterns and cognitive function. This study included 18 080 black and white participants aged 45 years and older from the REasons for Geographic And Racial Differences in Stroke (REGARDS) cohort. Principal component analysis on data from the Block98 FFQ yielded five dietary patterns: convenience, plant-based, sweets/fats, Southern, and alcohol/salads. Incident cognitive impairment was defined as shifting from intact cognitive status (score >4) at first assessment to impaired cognitive status (score ≤4) at latest assessment, measured by the Six-Item Screener. Learning, memory and executive function were evaluated with the Word List Learning, Word List Delayed Recall, and animal fluency assessments. In fully adjusted models, greater consumption of the alcohol/salads pattern was associated with lower odds of incident cognitive impairment (highest quintile (Q5) *v*. lowest quintile (Q1): OR 0·68; 95 % CI 0·56, 0·84; *P* for trend 0·0005). Greater consumption of the alcohol/salads pattern was associated with higher scores on all domain-specific assessments and greater consumption of the plant-based pattern was associated with higher scores in learning and memory. Greater consumption of the Southern pattern was associated with lower scores on each domain-specific assessment (all *P* < 0·05). In conclusion, dietary patterns including plant-based foods and alcohol intake were associated with higher cognitive scores, and a pattern including fried food and processed meat typical of a Southern diet was associated with lower scores.

As average life expectancy continues to increase due to progressive advances in the prevention and treatment of chronic disease^(^^1^^)^, Americans are enjoying the benefits of a prolonged life while simultaneously discovering the consequences of an ageing population, particularly those related to a decline in cognitive function. In the USA, where the prevalence of Alzheimer's disease and other dementias is expected to triple by 2050^(^^2^^)^, identifying modifiable risk factors that contribute to cognitive function is a growing area of research and could aid in the preservation of quality of life in older ages.

Several studies have evaluated the contributions of specific foods and nutrients to cognitive function, and some evidence suggests that regular consumption of foods such as fatty fish, nuts and berries, among others, could be related to more favourable cognitive outcomes^(^^3^^–^^8^^)^. Although these studies have provided valuable information, one limitation is that this type of approach does not accurately reflect the way people consume foods. Rather than individual foods or nutrients, people generally consume a combination of foods in meals that fall within an overall dietary pattern. By taking advantage of the potential interactions and collective effects of multiple foods, dietary patterns may be more predictive of cognitive function than foods or nutrients in isolation^(^^9^^)^.

Previous studies using investigator-defined dietary pattern analysis have demonstrated that adherence to a Mediterranean diet pattern or the Mediterranean–DASH Intervention for Neurodegenerative Delay (MIND) dietary pattern was associated with a reduced risk of cognitive impairment and slower cognitive decline^(^^10^^–^^12^^)^. However, these dietary patterns are typically defined *a priori* by investigators and may not reflect true or realistic patterns of food consumption within a population. As an alternative, this study aimed to use principal component analysis (PCA) to employ an empirical approach to identify dietary patterns that may more accurately represent the dietary habits of our sample. A number of studies have used similar methodology but have possessed smaller sample sizes generalisable to only one race^(^^13^^–^^19^^)^. This study utilised the REasons for Geographic And Racial Differences in Stroke (REGARDS) cohort, which consists of 30 239 black and white participants dispersed throughout continental USA. Within the REGARDS cohort, we have previously identified five dietary patterns^(^^20^^)^: convenience, plant-based, sweets/fats, Southern, and alcohol/salads. The objective of this study was to examine the associations between empirically derived dietary patterns, incident cognitive impairment, and cognitive performance on three domain-specific assessments in a large cohort of black and white adults over the age of 45 years. Our hypotheses were that the convenience, sweets/fats and Southern dietary patterns would be associated with poorer cognitive outcomes and that the plant-based and alcohol/salads dietary patterns would be associated with more favourable cognitive outcomes.

## Experimental methods

### Study sample

The REGARDS study is a national cohort of 30 239 community-dwelling black and white participants aged 45 years and older at baseline. Participants were recruited from 2003–2007 using lists purchased from Genesys, Inc. that were selected to oversample both black Americans and residents of the region of the Southeast USA known as the stroke belt. Upon entry into the study, the full cohort of participants had a mean age of 64·8 years (ranging from 45 to 98 years) and was approximately 42 % black, 55 % female and 56 % living in the stroke belt. Exclusion criteria included belonging to a race other than white or black, currently undergoing active treatment for cancer or another medical condition that could affect long-term study participation, nursing home residence or the inability to communicate in English.

The data from this analysis were collected primarily by using computer-assisted telephone interviewing and an in-home examination. The initial telephone call collected data regarding demographics, socio-economic status and medical history. An in-home examination by a trained medical professional followed where anthropometrics, blood and urine samples, blood pressure measurements and an electrocardiogram were collected. Additionally, several self-administered questionnaires were left with the participant to complete and mail back to the REGARDS coordinating centre. Additional details of the study design have been described in depth elsewhere^(^^21^^)^. This study was conducted according to the guidelines laid down in the Declaration of Helsinki and all procedures involving human subjects were approved by the institutional review boards of all participating institutions. Written informed consent was obtained by all participants included in this study.

### Assessment of dietary patterns

Dietary intake of the participants was assessed using the Block98 food frequency questionnaire (Block98 FFQ), which aims to assess usual dietary intake over the past year by including questions about both frequency and portions of various foods. The Block98 FFQ assesses food frequency by asking participants how often they consume each food item, with the following possible answers: never, a few times per year, once per month, 2–3 times per month, once per week, 2 times per week, 3–4 times per week, 5–6 times per week, or every day. The Block98 FFQ additionally assesses the usual quantity of food consumed by asking the participant how much of each food item they consume, on average, each time they consume that food item. For foods consumed in individual units such as eggs, bacon and doughnuts, participants were asked to choose the number that represents the usual quantity of that food they consume (i.e. 1 egg, 2 eggs, 3 eggs, or 4 eggs). To help estimate usual quantity consumed for other items such as spinach or ice cream, participants were provided a photograph that illustrated several common portions of foods (¼ cup, ½ cup, 1 cup, or 2 cups of foods on plates or ½ cup, 1 cup, or 2 cups of foods in bowls). Block FFQ have been previously validated using multiple food records^(^^22^^–^^24^^)^. The FFQ were left with the participant during the in-home examination, mailed back by the participant to the REGARDS coordinating centre, and sent to NutritionQuest for analysis.

The dietary patterns used in these analyses were derived previously^(^^20^^)^ and have been associated with incident stroke^(^^25^^)^, incident coronary artery disease^(^^26^^)^, sepsis^(^^27^^)^ and progression to end-stage renal disease in individuals with chronic kidney disease^(^^28^^)^. The 107 food items from the FFQ were combined into fifty-six food groups for use in PCA. Using a random split-sample technique to ensure validity and replication of the patterns, PCA with varimax rotation was utilised in the first half of the sample. Factor solutions were examined for interpretability and separate PCA were conducted to test for congruence by region, sex and race. Congruence coefficients were obtained to examine whether the dietary patterns could represent the entire sample or should be derived separately for these subgroups. In the second half of the sample, a confirmatory factor analysis including only the food groups with absolute value loadings ≥0·20 was used to independently validate the results from the PCA and test for model fit. After considering the screen test using eigenvalues >1·5 and examining the congruence coefficients to achieve optimal congruence across region, sex and race subgroups, this analysis retained five factors, and a final PCA with varimax rotation was performed in the full sample. Factor scores were calculated for each participant for each dietary pattern by multiplying the factor loading of each food group by each participant's average consumption of each food group.

The five dietary patterns were named according to the types of foods that loaded highly in each of them. Factor one was named the convenience pattern and consisted of mixed dishes with meat, pizza, Chinese food and Mexican dishes; factor two was named the plant-based pattern and consisted of vegetables, fruits, fish and beans; factor three included high factor loadings for miscellaneous sugars, desserts, candy, sweetened breakfast foods and added fats and was named the sweets/fats pattern; factor four was named the Southern pattern because of its high loadings of added fats, fried food, eggs and egg dishes, organ meats, processed meats and sugar-sweetened beverages; and factor five was named the alcohol/salads pattern and loaded highly in green-leafy vegetables, tomatoes, salad dressing, wine and liquor. Full factor loadings for each pattern are shown in Supplementary Table S1. In total, the five dietary patterns explained approximately 24 % of the total variance in dietary intake in the REGARDS sample, which is similar to other dietary pattern analyses reported in the literature^(^^29^^)^. The amount of variance explained by each dietary pattern is as follows: convenience, 8·7 %; plant-based, 5·9 %; sweets/fats, 3·6 %; Southern, 3·0 %; and alcohol/salads, 2·7 %.

### Assessment of cognitive function

Given the large, nationwide distribution of the REGARDS study, the cognitive assessment of its participants required a brief assessment that was able to be delivered over telephone. Beginning December 2003, the Six-Item Screener (SIS)^(^^30^^)^ was administered during baseline telephone calls and subsequently in annual intervals. The SIS is a brief screening assessment that consists of a three-item word recall and three-items pertaining to temporal orientation. Intact cognitive function was defined as having a score of 5 or 6 correct, and incident cognitive impairment was defined as shifting from intact cognitive function on the first cognitive assessment to impaired cognitive function (a score ≤4) on the most recent cognitive assessment^(^^30^^)^. Using a combined endpoint of dementia and mild cognitive impairment in a diverse community-based sample, the cut-point of 4 or fewer correct on the SIS has a sensitivity and specificity of 74 and 80 %, respectively^(^^30^^)^.

In January 2006, a three-test battery of domain-specific assessments was administered by telephone to participants and has been subsequently administered every 2 years. To assess verbal learning and memory domains, the Word List Learning (WLL) and Word List Delayed Recall (WLDR) from the Consortium to Establish a Registry for Alzheimer's Disease (CERAD) battery^(^^31^^)^ were administered. These assessments involved a set of three learning trials of a list of ten words followed by a 5 min delay that preceded a free recall trial. For WLL, the scores from the three trials were summed and produced a score ranging from zero to 30. For the WLDR, the score reflects the number of words the participant could recall after a 5 min delay and ranges from zero to 10. For both measures, repetitions and intrusions were excluded, and a procedure was implemented to exclude non-standard performance patterns (occurring in <2 % of the sample). To assess executive function, the Animal Fluency Test (AFT)^(^^31^^)^ was administered. This test required participants to name as many animals as they could in 1 min, yielding a raw score that was then adjusted for repetitions and intrusions.

For this analysis, the primary outcome is incident cognitive impairment as measured by the SIS. Due to the limited number of participants with multiple assessments for the domain-specific cognitive measures, we will be examining cross-sectional cognitive performance by including only the first measure of the WLL, WLDR and AFT assessments for each participant who possessed dietary data and were free of stroke at baseline.

### Covariate assessment

Age (continuous in years), race (dichotomous: black/white), sex (dichotomous: male/female), region of residence (categorical: stroke-belt, stroke-buckle, non-belt or buckle), income (categorical: <$20 000/year, $20 000–$34 999/year, $35 000–$74 999/year, >$75 000/year, and refused to provide income information) and education (categorical: less than high school, high school graduate, some college, college graduate) were self-reported at baseline. Total energy intake (continuous in kcal) was estimated from the FFQ administered at baseline. Height and weight were obtained from the in-home examination and used to calculate BMI (continuous in kg/m^2^). Physical activity defined by exercise frequency (categorical: none, 1–3 times/week, 4+ times/week) and smoking status (categorical: current, past, never) were self-reported at baseline. History of heart disease (dichotomous: yes/no) was defined as self-reported myocardial infarction, coronary artery bypass graft, angioplasty, stenting or evidence of myocardial infarction from an electrocardiogram performed during the in-home examination. Participants were defined as hypertensive (dichotomous: yes/no) if systolic blood pressure was ≥140 mmHg or diastolic blood pressure was ≥90 mmHg or if they self-reported current medication use to control blood pressure. Diabetes status (dichotomous: yes/no) was defined as having a fasting glucose ≥7 mmol/l (≥126 mg/dl) or non-fasting blood glucose ≥11 mmol/l (≥200 mg/dl) or if the participant reported taking medication or insulin for the management of diabetes. Depressive symptoms (continuous in Center for Epidemiological Studies – Depression four-item version (CESD-4) item score units) were evaluated at baseline over the telephone using the CESD-4^(^^32^^)^.

### Statistical analysis

Likelihood-ratio χ^2^ tests and *t* tests were used to calculate unadjusted means of demographic characteristics by quintile of each dietary pattern. Logistic regression was utilised to examine the relationship between quintiles of dietary pattern scores and odds of incident cognitive impairment via the SIS. Three models incrementally adding covariates were evaluated in this analysis. Model 1 included adjustment for age, race, sex, region and total energy intake. Model 2 additionally adjusted for socio-economic variables previously shown to affect cognitive function: income and education. Model 3 added adjustments for other known cognitive risk factors: physical activity, smoking status, BMI, hypertensive status, diabetes status, history of CVD and depressive symptoms. Participants with non-missing values for all covariates were included in each model, resulting in 0, 0·03 and 7·5 % missing data for each model, respectively. Effect modification for race and sex was examined by placing an interaction term in the model for each pattern. Tests for linear trend across quintiles of dietary patterns were evaluated by including each dietary pattern in quintiles as a continuous, ordinal variable in each model. Multiple regression was utilised to evaluate mean differences between quintiles of dietary patterns and each of the three domain-specific cognitive assessments, including all of the covariates listed previously to adjust for confounding. Analyses for the AFT also included a covariate to adjust for the participants who received assistance from someone in their home environment or was given a disallowed prompt by the interviewer (about 3·4 % of the sample).

## Results

Of the 30 239 original REGARDS participants, 72 % of the cohort returned a usable FFQ. This analysis excluded participants not returning a usable FFQ (*n* 8603), defined as the following: did not return a FFQ (17 % of full sample), returned a blank FFQ (3 %), possessing >15 % missing data on FFQ (5 %), or estimated to consume implausible energy intakes on FFQ (3 %)^(^^20^^,^^26^^)^.

Of the dietary subsample of REGARDS, participants were excluded if they did not possess at least two SIS assessments (*n* 1191) or were cognitively impaired at baseline (*n* 1447). Participants who self-reported history of stroke at baseline or had an incident stroke prior to first cognitive assessment (*n* 905) were also excluded. Finally, participants lacking an in-home medical assessment were excluded from these analyses (*n* 13). These exclusions resulted in a final sample of 18 080 participants. Additionally, cross-sectional analysis of cognitive performance on domain-specific assessments was performed in REGARDS participants possessing at least one WLL, WLDR and AFT assessment, dietary data and no history of stroke prior to cognitive assessment (*n* 14 247). Participants excluded from the longitudinal analyses were more likely than included participants to be older, male, black, less educated, and have lower income. Excluded participants were also more likely to report no weekly physical activity, currently smoke, have a higher BMI, have a history of hypertension, diabetes and CVD, and exhibit more depressive symptoms.

Descriptive statistics of participants who were included in this analysis are provided in Table 1. Compared with participants in the lowest quintile (Q1), participants in the highest quintile (Q5) of consumption of the convenience pattern tended to be younger, white, male, live outside the stroke belt, and have a higher income and a higher education level. Participants in Q5 of the plant-based pattern tended to be older, a higher proportion black, female, and possess a higher education level than participants in Q1. Participants in the Q5 of the sweets/fats pattern tended to be more white, male, stroke-belt residents, with a lower income and education than participants in Q1. For the Southern pattern, Q5 participants were more likely to be black, male, residing in the stroke-belt, and possess a lower income and education level than participants in Q1. Finally, participants in Q5 of the alcohol/salads pattern tended to be more likely to be younger, white, male, residing outside the stroke-belt, with a higher income and education level.

**Table 1.** Baseline characteristics by quintile of dietary pattern in the REasons for Geographic And Racial Differences in Stroke (REGARDS) cohort 2003–2014 (Number of participants and percentages, mean values and standard deviations)

	Convenience				Plant-based				Sweets/fats				Southern				Alcohol/salads			
	Q1		Q5		Q1		Q5		Q1		Q5		Q1		Q5		Q1		Q5	
	*n*	%	*n*	%	*n*	%	*n*	%	*n*	%	*n*	%	*n*	%	*n*	%	*n*	%	*n*	%
Age (years)																				
Mean	66·9		61·5		61·9		65·3		63·6		63·7		64·2		63·1		65·8		63·1	
sd	9·0		8·5		8·8		8·9		8·5		9·3		9·1		8·8		9·4		8·7	
Race																				
Black	1613	44·6	731	20·2	887	24·5	1296	35·8	1578	43·6	937	25·9	276	7·6	2146	59·4	1731	47·9	613	17·0
White	2003	55·4	2885	79·8	2729	75·5	2320	64·2	2038	56·4	2679	74·1	3340	92·4	1470	40·7	1885	52·1	3003	83·1
Sex																				
Male	1198	33·1	1995	55·2	1935	53·5	1281	35·4	1304	36·1	1767	48·9	1315	36·4	1961	54·2	1264	35·0	1874	51·8
Female	2418	66·9	1621	44·8	1681	46·5	2335	64·6	2312	63·9	1849	51·1	2301	63·6	1655	45·8	2352	65·0	1742	48·2
Region																				
Stroke belt	1416	39·2	1145	31·7	1245	34·4	1198	33·1	1096	30·3	1356	37·5	1020	28·2	1469	40·6	1263	34·9	1083	30·0
Stroke buckle	812	22·5	680	18·8	775	21·4	792	21·9	837	23·2	843	23·3	695	19·2	906	25·1	868	24·0	740	20·5
Non-belt	1388	38·4	1791	49·5	1596	44·1	1626	45·0	1683	46·5	1417	39·2	1901	52·6	1241	34·3	1485	41·1	1793	49·6
Total energy intake (kcal)																				
Mean	1436		2347		1568		2088		1287		2429		1719		2188		1649		2031	
sd	573		734		686		741		556		715		624		789		745		726	
Total energy intake (kJ)																				
Mean	6008		9820		6561		8736		5385		10163		7192		9155		6899		8498	
sd	2397		3071		2870		3100		2326		2992		2611		3301		3117		3038	
Income																				
<$20 000/year	685	18·9	399	11·0	502	13·9	507	14·0	522	14·4	561	15·5	268	7·4	815	22·5	881	24·4	246	6·8
$20 000–$34 999/year	996	27·5	718	19·9	844	23·3	800	22·1	795	22·0	900	24·9	658	18·2	984	27·2	991	27·4	654	18·1
$35 000–$74 999/year	1080	29·9	1225	33·9	1188	32·9	1193	33·0	1087	30·1	1223	33·8	1251	34·6	1054	29·2	945	26·1	1263	34·9
>$75 000/year	411	11·4	909	25·1	696	19·3	678	18·8	776	21·5	549	15·2	969	26·8	378	10·5	345	9·5	1073	29·7
Refused	444	12·3	365	10·1	386	10·7	438	12·1	436	12·1	383	10·6	470	13·0	385	10·7	454	12·6	380	10·5
Education																				
Less than high school	448	12·4	213	5·9	333	9·2	233	6·5	292	8·1	359	9·9	114	3·2	564	15·6	483	13·4	129	3·6
High school graduate	1000	27·7	791	21·9	1077	29·8	713	19·7	819	22·7	999	27·6	680	18·8	1122	31·0	1046	28·9	684	18·9
Some college	995	27·5	981	27·1	1027	28·4	979	27·1	1011	28·0	1034	28·6	906	25·1	1037	28·7	1003	27·8	921	25·5
College graduate	1171	32·4	1631	45·1	1177	32·6	1690	46·8	1493	41·3	1222	33·8	1915	53·0	892	24·7	1083	30·0	1880	52·0

Q1, lowest quintile; Q5, highest quintile.

Of the 18 080 participants included in this analysis, 1486 cases of incident cognitive impairment were identified over an average follow up of 6·8 years. Odds of incident cognitive impairment by quintile of each dietary pattern are displayed in Table 2. After adjustment for demographic factors and total energy intake, participants in the highest quintile of the Southern dietary pattern had higher odds of incident cognitive impairment (Q5 *v*. Q1: OR 1·46; 95 % CI 1·19, 1·78; *P* for trend ≤ 0·0001) compared with participants in the lowest quintile. Additionally, participants in the highest quintile of the plant-based and alcohol/salads dietary patterns had lower odds of incident cognitive impairment (plant-based – Q5 *v*. Q1: OR 0·81; 95 % CI 0·67, 0·98; *P* for trend = 0·02; alcohol/salads – Q5 *v*. Q1: OR 0·65; 95 % CI 0·54, 0·79; *P* for trend ≤0·0001). After further adjustment for socio-economic status and other cognitive risk factors, the observed associations with the plant-based and Southern patterns were attenuated and no longer statistically significant, but the association with the alcohol/salads pattern remained (Q5 *v*. Q1: OR 0·68; 95 % CI 0·56, 0·84; *P* for trend = 0·0005). No significant associations between the convenience and sweets/fats dietary patterns and incident cognitive impairment were observed, and tests for interactions by race and sex were non-significant for each pattern.

**Table 2.** Odds of incident cognitive impairment by quintile of dietary pattern in the REasons for Geographic And Racial Differences in Stroke (REGARDS) study 2003–2014 (*n* 18 080)* (Odds ratios and 95 % confidence intervals)

		Quintile 2		Quintile 3		Quintile 4		Quintile 5		
	Quintile 1	OR	95 % CI	OR	95 % CI	OR	95 % CI	OR	95 % CI	*P*_trend_
Convenience										
Impaired/total	386/3616	318/3616		321/3616		249/3616		212/3616		
Model 1	1	0·92	0·79, 1·09	1·05	0·89, 1·24	0·85	0·71, 1·02	0·85	0·69, 1·04	0·1
Model 2	1	0·95	0·80, 1·11	1·10	0·93, 1·29	0·88	0·74, 1·06	0·88	0·72, 1·09	0·25
Model 3	1	0·96	0·81, 1·14	1·06	0·89, 1·26	0·86	0·71, 1·03	0·87	0·70, 1·08	0·14
Plant-based										
Impaired/total	272/3616	298/3616		336/3616		288/3616		292/3616		
Model 1	1	0·89	0·74, 1·06	0·94	0·79, 1·12	0·80	0·66, 0·96	0·81	0·67, 0·98	0·02
Model 2	1	0·93	0·78, 1·11	1·00	0·84, 1·19	0·86	0·72, 1·04	0·91	0·75, 1·11	0·25
Model 3	1	0·92	0·77, 1·11	0·98	0·81, 1·18	0·87	0·71, 1·05	0·89	0·73, 1·10	0·23
Sweets/fats										
Impaired/total	271/3616	311/3616		305/3616		295/3616		304/3616		
Model 1	1	1·07	0·90, 1·28	1·06	0·89, 1·27	1·07	0·89, 1·29	1·23	1·00, 1·53	0·12
Model 2	1	1·05	0·88, 1·25	1·04	0·87, 1·25	1·03	0·85, 1·25	1·14	0·92, 1·41	0·38
Model 3	1	1·07	0·89, 1·28	1·05	0·87, 1·27	1·02	0·84, 1·25	1·19	0·95, 1·49	0·31
Southern										
Impaired/total	217/3616	254/3616		297/3616		348/3616		370/3616		
Model 1	1	1·03	0·85, 1·25	1·14	0·94, 1·38	1·29	1·07, 1·56	1·46	1·19, 1·78	<0·0001
Model 2	1	1·01	0·83, 1·22	1·09	0·90, 1·32	1·18	0·97, 1·43	1·23	1·00, 1·52	0·02
Model 3	1	0·97	0·79, 1·19	1·07	0·88, 1·31	1·16	0·95, 1·42	1·16	0·93, 1·45	0·05
Alcohol/salads										
Impaired/total	397/3616	343/3616		271/3616		270/3616		205/3616		
Model 1	1	0·94	0·80, 1·11	0·78	0·66, 0·93	0·83	0·70, 0·98	0·65	0·54, 0·79	<0·0001
Model 2	1	0·98	0·83, 1·15	0·83	0·70, 0·99	0·91	0·77, 1·08	0·76	0·62, 0·92	0·005
Model 3	1	0·94	0·80, 1·12	0·81	0·68, 0·97	0·88	0·73, 1·05	0·68	0·56, 0·84	0·0005

CESD-4, Center for Epidemiological Studies – Depression four-item version.

* Model 1 adjusts for demographic variables (age, race, sex, region and total energy intake). Model 2 additionally adjusts for socio-economic variables (income and education). Model 3 additionally adjusts for cognitive risk factors and co-morbidities (physical activity, smoking status, BMI, hypertensive status, diabetes status, history of CVD and score on the CESD-4).

**Fig. 1.**
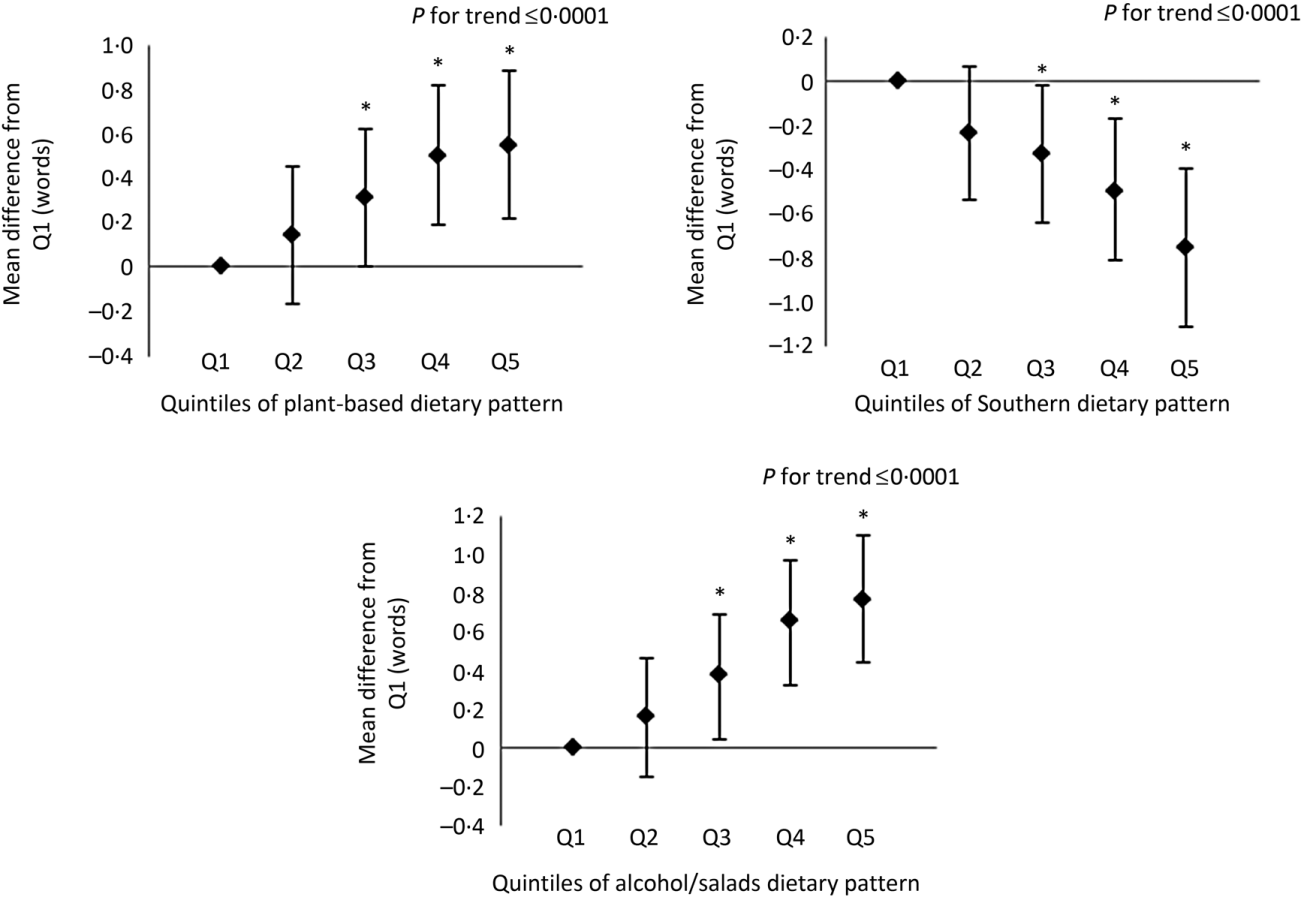
Multivariable-adjusted mean differences and 95 % confidence intervals on the Word List Learning assessment. Adjusted for age, race, sex, region, total energy intake, income, education, physical activity, smoking status, BMI, hypertensive status, diabetes status, history of CVD and depressive symptoms. Example interpretation: participants with factor scores in quintiles (Q) 3, 4 and 5 of the Southern dietary pattern scored significantly lower on the Word List Learning assessment than participants in Q1. * Mean differences were statistically significant (*P* < 0·05).

**Fig. 2.**
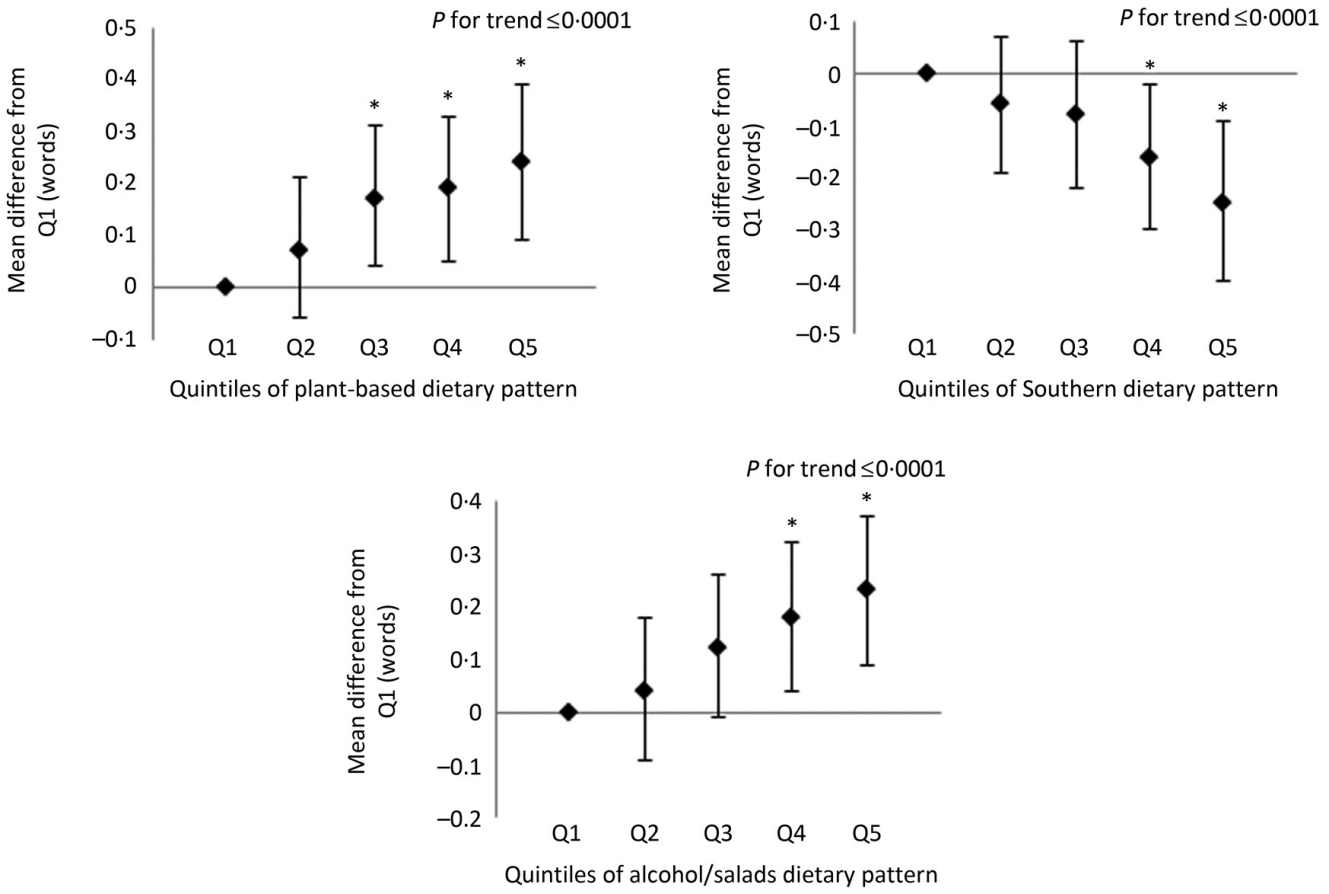
Multivariable-adjusted mean differences and 95 % confidence intervals on the Word List Delayed Recall assessment. Adjusted for age, race, sex, region, total energy intake, income, education, physical activity, smoking status, BMI, hypertensive status, diabetes status, history of CVD and depressive symptoms. Example interpretation: participants with factor scores in quintiles (Q) 4 and 5 of the Southern dietary pattern scored significantly lower on the Word List Delayed Recall assessment than participants in Q1. * Mean differences were statistically significant (*P* < 0·05).

**Fig. 3.**
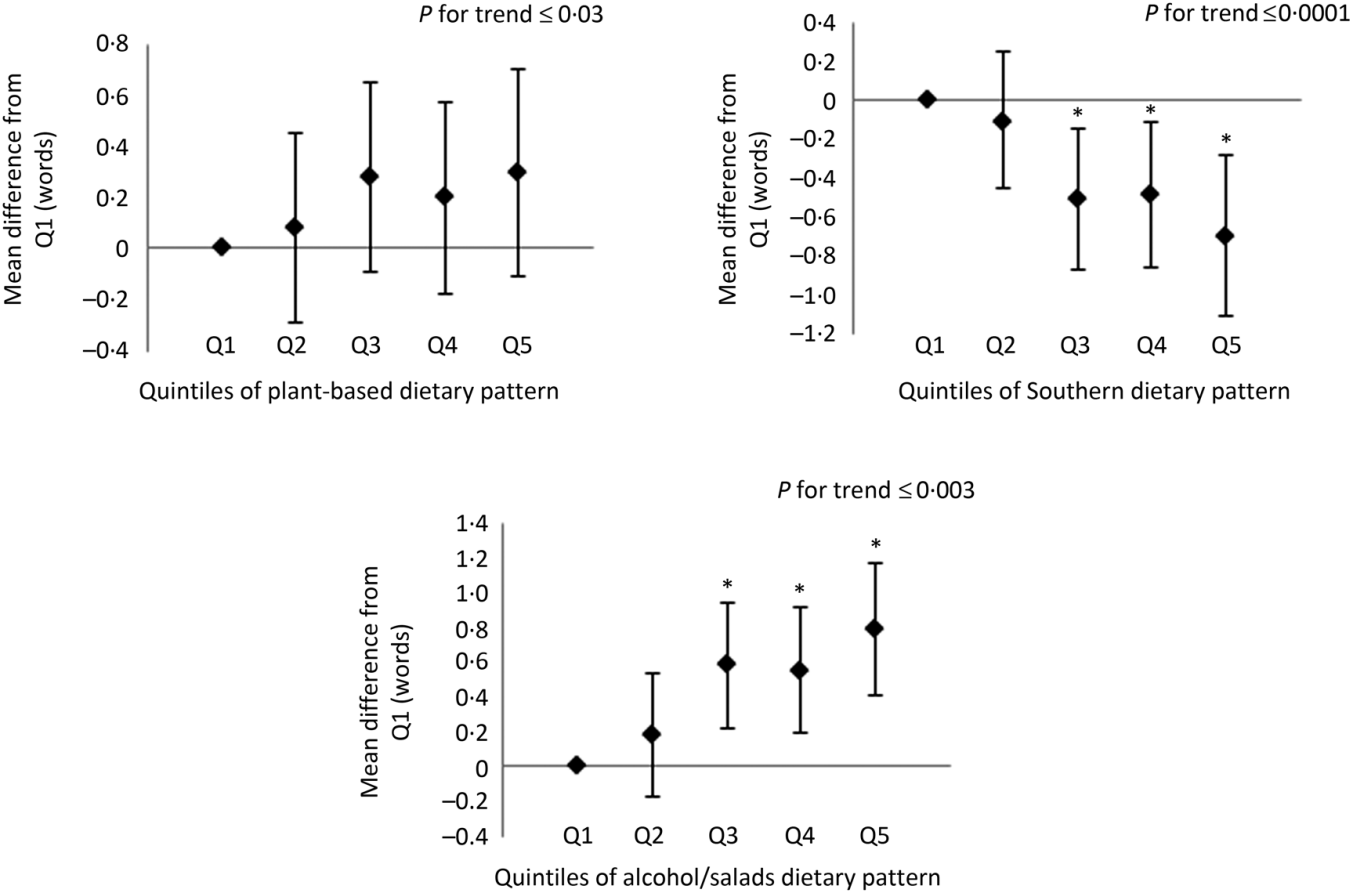
Multivariable-adjusted mean differences and 95 % confidence intervals on the Animal Fluency Test. Adjusted for age, race, sex, region, total energy intake, income, education, physical activity, smoking status, BMI, hypertensive status, diabetes status, history of CVD, depressive symptoms and disallowed help/prompting. Example interpretation: participants with factor scores in quintiles (Q) 3, 4 and 5 of the Southern dietary pattern scored significantly lower on the Animal Fluency Test than participants in Q1. * Mean differences were statistically significant (*P* < 0·05).

In the assessments of learning, memory and executive function, participants in the highest quintile of the alcohol/salads patterns had higher scores on all measures of cognitive function compared with participants in the lowest quintile (Figs 1–3). Likewise, participants with the highest consumption of the plant-based pattern scored higher on the WLL and WLDR assessments compared with participants with the lowest consumption (Figs 1 and 2). There were no differences in scores on the AFT between the extreme quintiles of the plant-based pattern, but a significant linear trend was observed (see Fig. 3). Additionally, scoring in the highest quintile of the Southern dietary pattern was associated with significantly lower scores in the learning, memory and executive function domains. Scoring in the highest quintile of the convenience dietary pattern was also associated with higher performance on the WLL (*P* < 0·05). No other differences were detected on any domain-specific assessments between any of the quintiles for the convenience and sweets/fats patterns, although a significant linear trend was observed on the WLL for the convenience and sweets/fats patterns and for the convenience pattern on the AFT. Domain-specific results for the plant-based, Southern and alcohol/salads patterns are displayed in Figs 1–3, and further details are provided in Supplementary Table S2.

## Discussion

In this study of 18 080 black and white participants aged 45 years and older, we found that greater consumption of the alcohol/salads dietary pattern was associated with lower odds of incident cognitive impairment and higher performance on several cognitive measures assessing learning, memory and executive function. Additionally, greater consumption of a plant-based dietary pattern was associated with higher cognitive performance while greater consumption of a Southern dietary pattern was associated with lower cognitive performance on these domain-specific measures. Our findings strengthen the body of literature that collectively suggests that dietary patterns may have an impact on cognitive function, and this particular study provides a unique perspective by utilising empirically derived dietary patterns in a large, diverse sample of black and white adults living throughout the country.

As expected, greater consumption of the plant-based dietary pattern that loaded highest in many different types of vegetables, fruits and legumes was associated with higher cognitive performance on the WLL and WLDR assessments. This is consistent with previous studies, both cross-sectional and longitudinal, that have demonstrated an association between higher levels of fruit or vegetable intake and more favourable cognitive outcomes^(^^33^^–^^36^^)^. Many researchers have hypothesised that this observation could be related to higher intakes of fruits and vegetables contributing to higher levels of antioxidants, resulting in lower levels of oxidative stress. In a cross-sectional study of 193 healthy adults aged 45–102 years, Polidori *et al*.^(^^34^^)^ tested this hypothesis and found that adults who reported consuming higher intakes of fruits and vegetables had higher cognitive performance, higher levels of circulating antioxidant micronutrients, and lower levels of oxidative stress biomarkers compared with adults consuming lower amounts of fruits and vegetables. Additionally, higher fruit and vegetable intake has been associated with lower blood pressure^(^^37^^)^ and CVD incidence^(^^38^^)^, which are both known risk factors for cognitive impairment^(^^39^^,^^40^^)^ and may be mediating these associations despite attempts to adjust for confounding.

Interestingly, greater consumption of the alcohol/salads dietary pattern was associated with lower odds of incident cognitive impairment and higher cognitive performance on all cognitive assessments analysed in this study. Although previous studies utilising similar methodology have yielded dietary patterns comparable with the plant-based and Southern dietary patterns^(^^13^^,^^29^^,^^41^^)^, the alcohol/salads pattern appears to be unique to our cohort. We believe that the size and racial diversity of REGARDS participants geographically distributed throughout the USA provides the opportunity for our analysis to yield unique patterns that may not reflect the dietary patterns previously derived in participants of smaller, less diverse cohorts. The alcohol/salads pattern loaded highest on salad dressings/sauces and green leafy vegetables, and also contained a high factor loading for tomatoes. Green leafy vegetables and tomatoes are vegetables that are particularly high in antioxidants and could be contributing to cognitive function in similar ways described for the plant-based dietary pattern. This pattern also consisted of higher intakes of both wine and liquor. Several previous epidemiological studies have demonstrated an association between moderate alcohol consumption and more favourable cognitive outcomes, most citing the potential cardiovascular benefits of moderate alcohol consumption to be contributing to the increased cognitive performance^(^^42^^–^^45^^)^. It is also possible that the observed associations with the alcohol/salads pattern may be partially explained by reverse causation, especially the cross-sectional associations involving the domain-specific assessments. Several studies have reported that higher childhood cognitive ability is correlated with higher alcohol consumption in adulthood^(^^46^^,^^47^^)^. Since the REGARDS study does not possess cognitive data during childhood, we cannot exclude the possibility that the observed associations between the alcohol/salads dietary pattern and more favourable cognitive outcomes could at least partially be attributed to differences in childhood cognitive ability.

The Southern dietary pattern was associated with poorer cognitive performance on the WLL, WLDR and AFT assessments in this study. This was not surprising given the pattern's high factor loadings of fried food, processed meats, sugar-sweetened beverages and refined white bread. A similar ‘processed food pattern’ was identified by Torres *et al*.^(^^41^^)^ and also consisted of fried foods, processed meat and sugar beverages in 249 people aged 65–90 years with mild cognitive impairment. In that study, the highest intake of the processed food pattern was associated with the lowest cognitive performance on a global cognitive examination.

The results of this analysis must be interpreted with consideration of the study's limitations. Three of the five dietary patterns were associated with cognitive performance on multiple domain-specific assessments, but only the alcohol/salads pattern was associated with incident cognitive impairment on the SIS. This discrepancy may reflect a higher sensitivity of the domain-specific assessments to detect cognitive differences relative to the SIS. Additionally, through our use of multivariable modelling, we attempted to minimise the influence of several confounders on the associations between dietary patterns and cognitive function. Regardless of our efforts, the possibility of residual confounding still remains. The correlation between socio-economic status and cognition is well established, and several studies have reported attenuations in associations between dietary patterns and various cognitive outcomes after the adjustment of socio-economic measures^(^^13^^,^^48^^)^. However, it is notable that many of the associations between dietary patterns and cognitive function in this analysis remained significant even after adjustment for income and education. One final limitation is the possibility for recall bias to exist in the measurement of our dietary data by FFQ. It is reasonable to suggest that participants with lower cognitive function would have more difficulty providing accurate dietary data via recall of food frequency. However, we attempted to minimise the potential of recall bias by excluding participants with cognitive impairment at baseline from the longitudinal analysis of incident cognitive impairment.

Despite these limitations, we believe this study provides a unique perspective of the diet–cognition relationship in a very large cohort of geographically dispersed black and white Americans. Utilising empirically derived dietary patterns with no pre-specification of diet quality, we identified a plant-based and alcohol/salads dietary pattern associated with higher cognitive performance and a Southern dietary pattern associated with lower cognitive performance. Findings from this study, in conjunction with previous literature, could be used to develop interventions to maintain the cognitive function of older Americans.

## Supplementary material

For supplementary material accompanying this paper visit http://dx.doi.org/10.1017/jns.2016.27. click here to view supplementary material
