# Supplementary material for: Dietary patterns are associated with cognitive function in the REasons for Geographic And Racial Differences in Stroke (REGARDS) cohort
Source: J Nutr Sci. 2016 Sep 28;5:e38. doi: 10.1017/jns.2016.27 (PMC5048188; doi:10.1017/jns.2016.27)
Supplement: Supplementary file 1 [file S2048679016000276sup001.pdf]

Supplementary Table 1. Factor loadings for each dietary pattern derived in the REasons for Geographic And Racial Differences in Stroke (REGARDS) Cohort 2003-2014

	Convenience	Plant-based	Sweets/Fats	Southern	Alcohol/Salads
**Food group**					
100% fruit juice	-0.051	0.25	0.041	0.17	-0.17
Added fats	0.11	0.053	0.4	0.38	0.25
Beans	0.36	0.38	-0.0026	0.09	-0.13
Beer	0.14	-0.16	-0.1	0.11	0.23
Bread	0.11	-0.05	0.47	0.37	-0.07
Bread - whole grain	-0.0025	0.3	0.18	-0.098	0.07
Butter	0.017	-0.016	0.17	0.13	0.32
Candy	0.085	-0.038	0.4	-0.1	-0.072
Cereal	-0.012	0.38	0.074	0.049	-0.2
Cereal - high fiber	0.068	0.24	0.048	-0.25	-0.044
Chinese food	0.44	0.029	-0.04	-0.022	0.15
Chocolate	0.13	-0.079	0.46	-0.12	-0.013
Coffee	0.0084	-0.063	0.22	-16	0.3
Condiments	0.25	0.06	0.31	0.15	0.29
Dairy - high fat	0.18	-0.067	0.37	0.043	0.21
Dairy - low fat	0.079	0.2	0.042	-0.19	-0.012
Desserts	0.2	0.04	0.53	0.11	-0.17
Eggs and egg dishes	0.012	-0.0087	0.11	0.42	0.29
Fish	0.27	0.38	-0.11	0.067	0.21
Fried food	0.24	0.023	0.1	0.56	-0.0067
Fried potatoes	0.37	-0.13	0.28	0.16	0.066
Fruit	-0.065	0.58	0.0077	-0.095	-0.029
Liquor	0.05	-0.1	-0.14	0.096	0.31
Margarine	0.041	0.045	0.37	0.1	-0.035
Mexican dishes	0.48	-0.09	0.048	-0.067	0.1
Milk alternatives	-0.012	0.18	-0.073	-0.027	-0.02
Milk - high fat	-0.1	0.012	0.18	0.24	-0.052
Milk - low fat	0.1	0.16	0.032	-0.42	0.0015
Miscellaneous sugar	-0.11	0.0042	0.54	0.19	0.0008
Mixed dishes with meat	0.61	0.13	0.05	0.053	0.026
Nuts and seeds	0.1	0.26	0.19	-0.098	0.19
Organ meat	0.17	0.068	-0.062	0.47	-0.087
Pasta dishes	0.59	0.089	0.17	-0.029	0.026
Pizza	0.45	-0.18	0.2	-0.12	0.074
Potatoes	0.36	0.12	0.26	0.031	0.025
Poultry	0.29	0.31	-0.045	0.034	0.13
Processed meats	0.25	-0.061	0.26	0.45	0.22
Red meat	0.45	-0.077	0.18	0.26	0.26
Refined grains	0.31	0.17	0.2	0.2	-0.0016

Salad dressing/sauces	0.12	0.3	0.045	-0.13	0.55
Salty snacks	0.32	-0.072	0.3	0.081	0.1
Shell fish	0.28	0.09	-0.08	0.23	0.24
Soda	0.096	-0.23	0.15	0.24	0.022
Soup	0.44	0.32	-0.0092	0.03	-0.15
Sugar-sweetened beverages	-0.023	0.064	0.068	0.37	-0.15
Sweet breakfast foods	0.19	-0.028	0.39	0.13	-0.14
Tea	-0.072	0.091	0.31	-0.024	0.054
Vegetable - cruciferous	0.067	0.59	-0.053	0.11	0.062
Vegetable - dark yellow	0.0098	0.41	0.055	0.13	-0.17
Vegetable - green leafy	0.16	0.49	-0.077	-0.22	0.48
Vegetable - other	0.052	0.48	0.041	-0.04	0.039
Vegetable - tomato	0.015	0.32	-0.026	0.018	0.27
Vegetable mixed dishes	0.35	0.31	-0.033	0.13	-0.25
Water	-0.093	0.32	-0.056	-0.024	0.086
Wine	0.062	0.021	-0.14	-0.14	0.36
Yogurt	0.075	0.31	0.035	-0.25	-0.04

Supplementary Table 2. Least squares means and mean differences between quintiles of dietary pattern adherence on the Word List Learning, Word List Delayed Recall, and Animal Fluency Test in the REasons for Geographic And Racial Differences in Stroke (REGARDS) Cohort 2003-2014

		Word List Learning			Word List Delayed Recall			Animal Fluency Test		
		**LS mean**	**Mean difference**	**P value**	**LS mean**	**Mean difference**	**P value**	**LS mean**	**Mean difference**	**P value**
**Convenience**										
	Q1	16.11	0	N/A	6.019	0	N/A	15.39	0	N/A
	Q2	16.14	0.037	1	6.088	0.068	0.63	15.32	-0.07	0.98
	Q3	16.34	0.23	0.27	6.088	0.069	0.63	15.56	0.17	0.73
	Q4	16.34	0.23	0.28	6.098	0.079	0.52	15.56	0.17	0.74
	Q5	16.48	0.37	0.03	6.1	0.085	0.56	15.71	0.32	0.23
**Plant-based**										
	Q1	15.98	0	N/A	5.95	0	N/A	15.33	0	N/A
	Q2	16.12	0.14	0.73	6.02	0.074	0.55	15.41	0.079	0.98
	Q3	16.29	0.31	0.05	6.12	0.17	0.005	15.61	0.28	0.24
	Q4	16.48	0.5	0.0001	6.14	0.19	0.0015	15.52	0.2	0.62
	Q5	16.53	0.55	<0.0001	6.18	0.24	<0.0001	15.63	0.3	0.26
**Sweets/Fats**										
	Q1	16.27	0	N/A	6.05	0	N/A	15.42	0	N/A
	Q2	16.45	0.18	0.49	6.14	0.089	0.36	15.53	0.12	0.91
	Q3	16.3	0.027	1	6.06	0.0041	1	15.39	-0.027	1
	Q4	16.28	0.004	1	6.1	0.043	0.91	15.69	0.27	0.3
	Q5	16.01	-0.26	0.3	6.03	-0.02	1	15.42	0.0037	1
**Southern**										
	Q1	16.72	0	N/A	6.21	0	N/A	15.95	0	N/A
	Q2	16.48	-0.24	0.22	6.15	-0.059	0.75	15.94	-0.011	1
	Q3	16.39	-0.33	0.03	6.14	-0.075	0.55	15.49	-0.46	0.008
	Q4	16.23	-0.5	0.0003	6.06	-0.16	0.02	15.43	-0.52	0.002
	Q5	15.96	-0.76	<0.0001	5.97	-0.25	0.0002	15.18	-0.76	<0.0001
**Alcohol/Salads**										
	Q1	15.91	0	N/A	5.97	0	N/A	15.22	0	N/A

Q2	16.07	0.16	0.61	6.01	0.042	0.91	15.42	0.19	0.6
Q3	16.29	0.38	0.007	6.09	0.12	0.1	15.68	0.46	0.007
Q4	16.57	0.66	<0.0001	6.15	0.18	0.003	15.57	0.35	0.09
Q5	16.68	0.77	<0.0001	6.2	0.23	0.0001	15.63	0.41	0.04

LS means adjusted for age, race, sex, region, total energy intake, income, education, physical activity, smoking status, BMI, hypertensive status, diabetes status, history of CVD, and score on the CESD. LS means for the Animal Fluency Test also adjusted for disallowed help/prompting. Mean differences represent the mean difference in cognitive performance between participants in each dietary pattern quintile compared to participants in quintile 1.
